# Self-Powered Triboelectric Nanogenerator for Security Applications

**DOI:** 10.3390/mi14030592

**Published:** 2023-03-01

**Authors:** Prabavathi Munirathinam, Arunkumar Chandrasekhar

**Affiliations:** Nanosensors and Nanoenergy Lab, Sensor Systems Lab, Department of Sensors and Biomedical Technology, School of Electronic Engineering, Vellore Institute of Technology, Vellore 632014, Tamil Nadu, India

**Keywords:** sliding, triboelectric, self-powered, biomechanical energy, security

## Abstract

Valuable jewels, documents, and files left in hotel rooms by guests can be stolen at any time by an unauthorized person. This could have a serious psychological and economic impact on the guests. The house/hotel owners should make efforts to prevent theft from occurring. In this study, a self-powered sliding-mode triboelectric nanogenerator (TENG) is used as a sensor on a drawer. It is fixed to the side of the drawer and works in the lateral sliding mode. The electricity generated by the device during the push–pull action of the draw is ~125 V and F~12.5 µA. An analysis of the electrical performance was carried out using PET, paper, and nitrile as sliding materials. The electrical output from the device is used to notify the guest or hotel owner of any theft by an unidentified individual via Arduino and node MCU devices. Finally, this device can be helpful at night and can be extended using different materials.

## 1. Introduction

In the economic world, security measures are essential for avoiding theft. The loss of expensive items, currency, important documents, and unique items can have emotional and economic consequences. People often store their valuable possessions securely in banks or security devices. It would be helpful to inform the owner of these items when someone, including an unidentified person, attempts to access by the secure device using a self-powered and low-cost solution. Most electrical devices, such as washing machines, automobiles, refrigerators, automatic doors, and automated vehicles, are controlled by sensors. However, the lifespan of the batteries in these devices needs replacing. Many researchers have been employed to find solutions to this issue and it has been found that self-powered devices can overcome the complication of replacing batteries [1,2]. An energy-harvesting mechanism is a key tool for generating power in a self-powered device [3,4]. Triboelectric nanogenerator technology is a reliable harvesting technique that harvests energy from human motion or mechanical movement [5]. The physics of the TENG are based on contact electrification and electrostatic induction [6]. The triboelectric nanogenerators (TENG) design, together with its energy-harvesting mechanism, paved the way for the development of self-powered sensors in 2012 [7,8]. Even though many sensors exist, TENG-based sensors have tremendous advantages such as material flexibility, ease of fabrication, availability of materials, nano-sized compactness, low costs, a minimum frequency of operation, and a stable device lifetime to generate power [9,10,11].

Researchers have made many contributions to the development of security-related portable/wearable devices [12]. The materials are the major advantage in the fabrication of the TENG device, and the surface roughness or surface modifications help to tune the output of the TENG device [13,14,15]. The material exhibits the property of electron repulsion or attraction between its components. Combining the material with different polarities will increase the TENG’s output [16,17]. In a TENG, there are four modes: lateral sliding mode, vertical contact-separation mode, freestanding mode, and single electrode mode [18,19,20,21]. Each mode has numerous practical uses across various fields, including security applications such as vehicle alert systems that monitor tire movement and intrusion detection systems that use paint for traffic and security purposes [22,23,24,25,26].

Herein, we propose a device for security purposes that is fixed beneath a table draw [27]. The push–pull action of the table’s draw drives the TENG device in lateral sliding mode. The sliding motion of the TENG’s (SM-TENG) mechanical energy is from the table draw’s movement. The fabricated device is low-cost, simple, and designed with readily available materials. Kapton and aluminium are used as the bottom triboelectric layers. The device produces a maximum output voltage of 125 V from the hand-sliding movement and nitrile is used as the sliding material. The TENG device was combined with Arduino and node MCU devices to create a self-powered system, which was subsequently tested as a security system within a secret room in a table drawer. The tests of this system were successful. Accessing the drawer, triggered both an alarm sound and a notification through the use of a buzzer sound and a light-emitting diode (LED). The proposed work will be helpful for use at night as a security application.

## 2. Design and Experimental Section

Materials: The materials used for the fabrication are an acrylic sheet with dimensions of 5 cm × 15 cm × 3 mm, an aluminium electrode measuring 4 cm × 1.5 cm, a Kapton film measuring 5 cm × 15 cm, as well as rubbing materials such as nitrile, paper, and PET.

**Figure 1:**
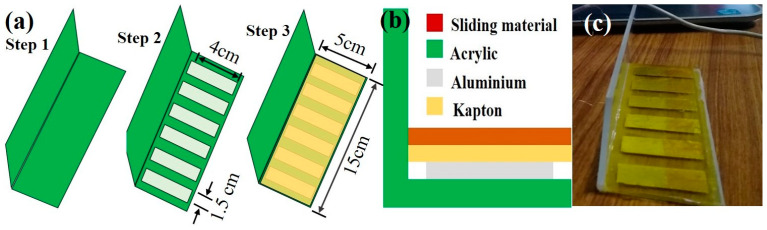
Fabrication and schematic illustration of the device: (**a**) The step-by-step fabrication process of the proposed device, (**b**) side view, (**c**) schematic illustration of the smart draw.

Fabrication process: Initially, the supporting surface of the device was fabricated; the acrylic sheet is strong enough to replace a table made from wood or metal. Here, we replaced the draw-like structure with an acrylic sheet in an L shape. The step-by-step attachment of the device’s top view is shown in Figure 1a. As can be seen in Figure 1b, copper wire was attached above the aluminium electrode and the Kapton film, which is the negative triboelectric layer, was placed on top of it. An exemplar model of the front view of the left side of the drawer with six segmented aluminium electrodes attached above the acrylic sheet as in Figure 1c. 

Electrical measurements and testing analysis: To measure the electrical output of the SM–TENG, an external sliding motion was applied by manually sliding a human hand. The sliding materials were pre-treated with heat at 50 °C (in a hot air oven) for 30 min to remove humidity. The output voltage and short-circuit current of the SM-TENG were measured using a Keysight Digital Storage Oscilloscope (DSOX2012A). Real-time data were measured after fixing the device to the table draw and the simulation was performed using COMSOL software. A self-powered analysis was performed using a hand-sliding motion to produce sufficient electricity to illuminate both an LED and an LCD.

## 3. Results and Discussion

**Figure 2:**
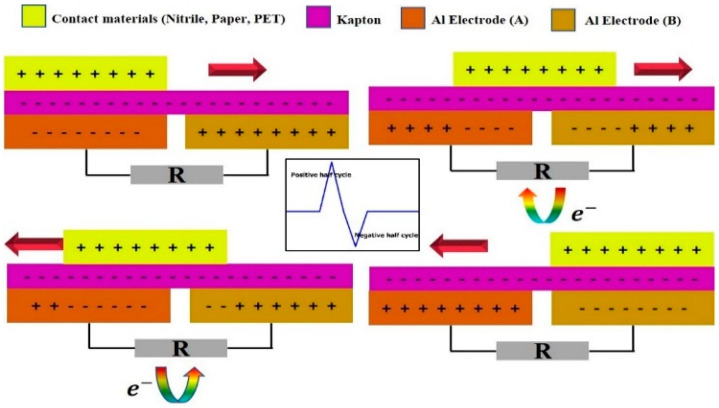
The working mechanism of the TENG device in lateral sliding mode.

**Figure 3:**
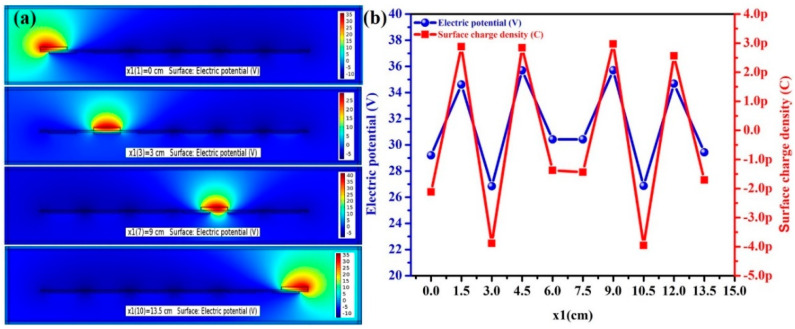
COMSOL simulation of the SM–TENG to (**a**) validate the working mechanism at x1 = 0, 3, 9, 13.5 cm, and (**b**) the electrical responses: electricity potential and surface charge density of the simulation.

The self-powered theft alarm system operates in the lateral sliding mode shown in Figure 2 [28]. The aluminium electrodes A and B are fixed to the acrylic sheet. In total, there are three pairs of TENGs. The electrons in Al electrode B flow towards Al electrode A through the external circuit sliding outwards, giving a positive half-cycle. For the negative half-cycle, the sliding materials must start sliding toward their original positions. Then, the electrons flow from Al electrode A to A1 electrode B. The sliding action of the draw creates electrical energy from the segments. If the number of sections increases, the electrical output increases based on the table’s size. As the positive triboelectric layer, nitrile, paper, and polyethylene terephthalate (PET) were chosen for testing and nitrile generated more electricity than the other materials. The charges are transferred from the positive material to the Kapton film when the sliding material, i.e., paper, PET, or nitrile (positive material), is moved outwards with the Kapton material (negative material) [29,30]. Hence, a positive half-cycle is generated and, likewise, a negative half-cycle was produced by sliding inwards. When the top layer starts sliding forward and backward as a result of the push–pull action of the draw, a potential difference is generated that results in the flow of electrons between the electrodes. The TENG returns to its starting position after one cycle of the AC output signal when the contact material fully slides out of the Kapton film [31]. A finite-element technique simulation using COMSOL Multiphysics was used to verify the functionality of the mechanism at various sliding distances with their related surface charge potentials, as shown in Figure 3. The sliding distance of 15 cm was based on the bottom layer. Figure 3a,b shows the electricity potential, with a maximum potential of 35 V, and the short-circuit transfer charge obtained was 2.9729 pC.

**Figure 4:**
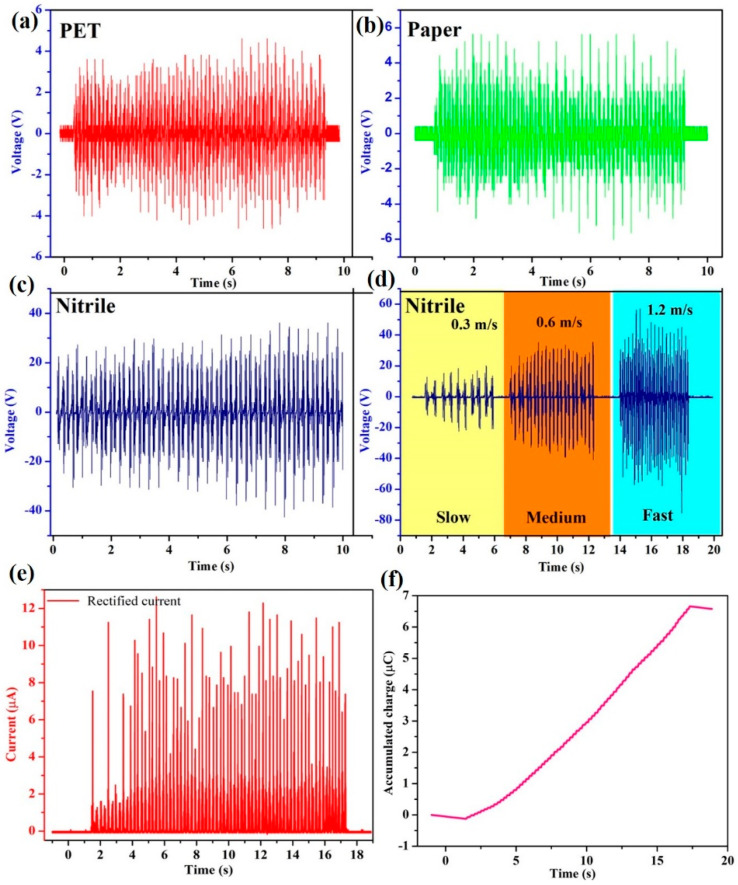
The electrical output of the SM–TENG: Voltage comparison with (**a**) PET, (**b**) paper, and (**c**) nitrile as sliding materials. (**d**) Open–circuit voltage under different sliding velocities with nitrile as sliding material. (**e**,**f**) Short–circuit current and accumulated charge.

The electrical output of the SM-TENG device using PET, paper, and nitrile as contact materials was tested. The contact materials were also used for testing the electrical output performance of the device, as shown in Figure 4a–c. The selected materials had a high positive surface charge compared to the Kapton film. These materials were chosen because they are affordable and widely available. Furthermore, if the material is damaged, it will not affect the function because it is easily replaceable. Kapton film is a negative material; it generates a charge when it interacts with other positively charged materials. Here, the SM-TENG provided a high output when it interacted with the positive triboelectric material nitrile. In the case of PET and paper, the minimum voltage was generated. The device generated a maximum electrical response of ~125 V and ~12.5 µA for nitrile, as shown in Figure 4d,e. Under different sliding velocities (0.3 m/s, 0.6 m/s, 1.2 m/s), nitrile was used as the sliding material throughout the analysis. The charge accumulated on the contact surface was proportional to the external motion. When the outward motion increased, it triggered the formation of a triboelectric charge and raised the charge accumulation. Figure 4f shows the charge accumulation on the triboelectric material [32].

**Figure 5:**
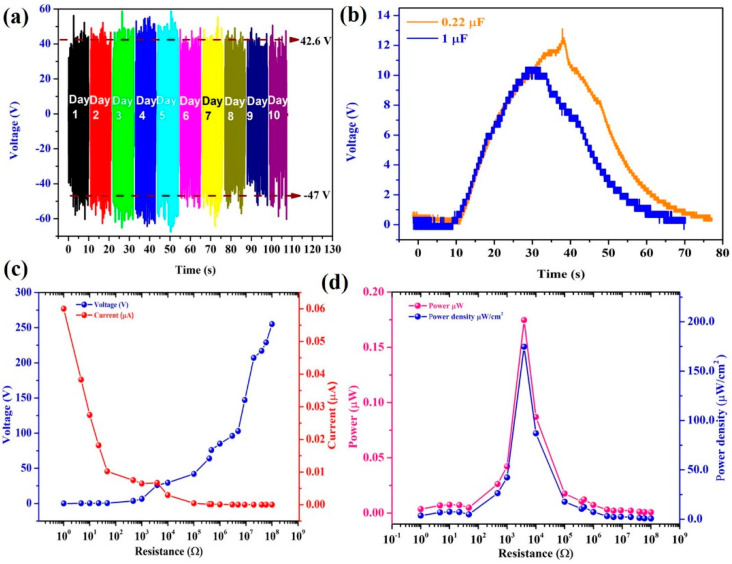
The electrical output of the SM–TENG: (**a**) The stability analysis of the SM-TENG device was recorded for 10 days, (**b**) the capacitor charging and discharging with 0.22 µF and 1 µF, (**c**) load-matching analysis of open-circuit voltage and short-circuit current, and (**d**) instantaneous peak power and power density of the SM-TENG device.

Real-time energy harvesting is the major goal of this device so it is essential to ensure its reliability and longevity, for which a 10-day stability test was conducted, as shown in Figure 5a. It was proven that the output performance was stable for the 10 days of continuous measurement. The output performance showed that the device was able to generate output for a long period, which is important in real-time applications. The stability of the device was also tested for a short duration of 100 s, which is shown in Figure S1 [33]. The charging and discharging abilities of the TENG device were validated using 1 µF and 0.22 µF capacitors and rectifiers (DF06G) [30]. The SM-TENG device charged the capacitor up to 13.111 V and 10.498 V, as shown in Figure 4b. Before incorporating the energy-harvesting device into a real-time application, the best load resistance for the TENG must be determined. To achieve this, we carried out external load resistance tests to determine the instantaneous peak power, load matching, and power density. The voltage in proportion to the load resistance is shown in Figure 5c. At a 3.8 K load resistance, a peak power of 0.17467 W and a power density of 174.669 mW/cm^2^ were obtained, as shown in Figure 5d. 

**Figure 6:**
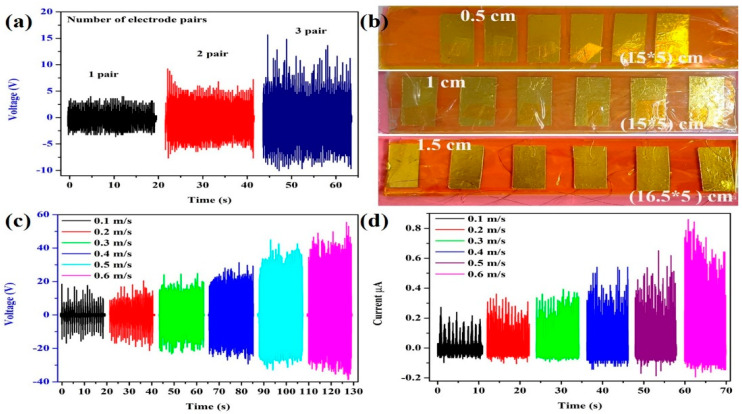
The design optimization analysis of the SM–TENG: (**a**) open-circuit voltage with different pairs of electrodes, (**b**) the device with different distances between the electrodes (distances of 0.5, 1, 1.5 cm) (corresponding output is shown in Figure S2), (**c**,**d**) open-circuit voltage and short-circuit current at different sliding velocities.

For device optimization, the device was tested with one set of electrodes, as shown in Figure 6a. The figure shows that the performance increased with an increase in the number of electrodes. The different distances between the electrodes, such as 0.5 cm, 1 cm, and 1.5 cm, as shown in Figure 6b, played a vital role in optimizing the device. The SM-TENG generated the best electrical output with a distance of 1 cm and the output is shown in Figure S2. The figure shows that the distance between the electrodes had a significant impact on the triboelectric performance. The optimum measurement for the proposed sensor was 1.5 cm × 1 cm. Figure 6c,d shows the output voltage and rectified short-circuit current of the SM-TENG with different sliding velocities. The output voltage reached ~42 V and the corresponding short-circuit current was 0.8 µA at a velocity of 0.6 m/s. This shows that by increasing the velocity from 0.1 m/s to 0.6 m/s, the open-circuit voltage and short-circuit current also increased [1,19,34]. 

**Figure 7:**
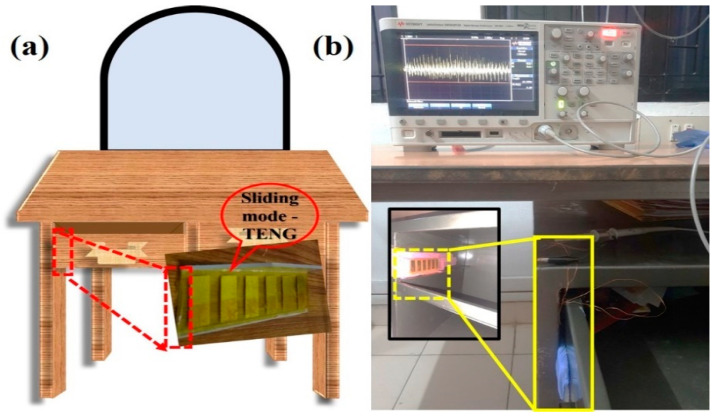
The device: (**a**) schematic of the energy-harvesting setup within the table’ draw, (**b**) the real-time demonstration of the SM-TENG device.

The main goal of this TENG (SM-TENG) technology was to scavenge energy. The graphical image of the device incorporated into a table’s draw is shown in Figure 7a. The real-time demonstration of the energy-harvesting device is shown in Figure 7b. The voltage response of the forward and backward sliding motion is shown in Figure S3.

**Figure 8:**
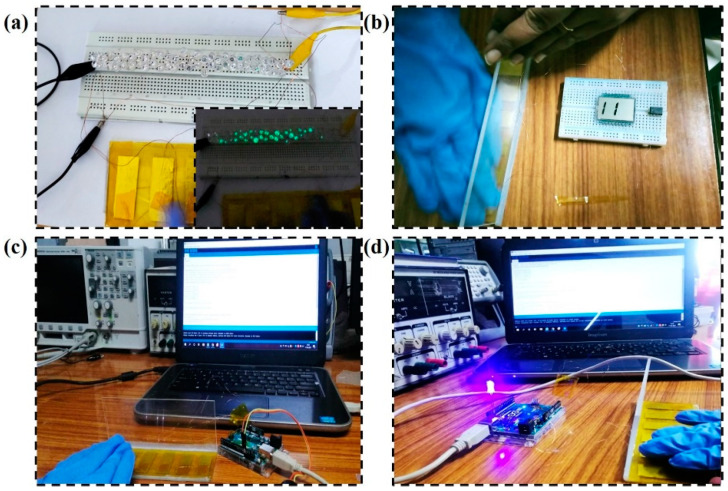
The setup shows the device with Arduino for theft protection: (**a**) LED illumination, (**b**) LCD displaying the number 11 when the draw slides, (**c**) the buzzer alert, and (**d**) the message on the monitor screen.

We used the SM-TENG device to drive low-power electronic devices such as LED and LCD. The SM-TENG was connected to small, green LEDs (light-emitting diodes), and, using nitrile as a sliding material, the output voltage was transferred to the LEDs to illuminate them, as shown in Figure 8a (the glowing LED is shown in the inset in Figure 8a). The LCD (liquid crystal display) was also connected and displayed the number 11, as shown in Figure 8b. The LED and LCD were illuminated with high intensity, which proved that without external batteries, the device was able to operate low-power electronic devices.

**Figure 9:**
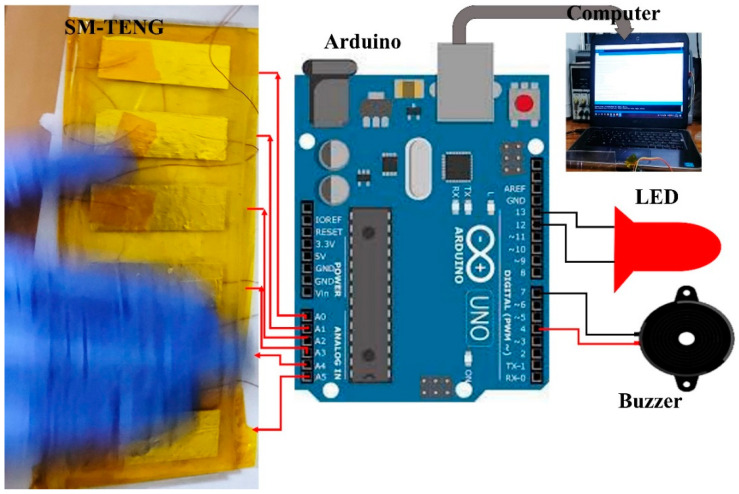
The setup shows the connection between the device, Arduino board, LED, buzzer, and computer.

Furthermore, the theft alarm system was successfully tested. When the drawer was opened, the TENG device generated an electrical signal. The Arduino board actuated the alarm sound and displayed a message on the computer screen, as shown in Figure 8c,d. The table draw sent a signal to the Arduino board and node MCU integrated into the device. The connection between the device, Arduino board, computer, LED, and buzzer for the security application is shown in Figure 9. We believe that the self-powered theft alarm system concept is innovative and in addition, the images below show how to commercialize this device as a scale-up product. 

## 4. Conclusions

In this paper, a sliding-mode self-powered anti-theft alarm system was created. The fabrication of device is simple and easy to fabricate with low costs. The materials used in this work are simple and readily available in stationery shops. A Kapton film was used as the negative triboelectric layer and an aluminium electrode were placed beneath it. The working mechanism of the device was a lateral sliding mode. Various sliding materials, including PET, paper, and nitrile, were tested for durability as the positive triboelectric layer. Analyses were carried out using various materials to analyze the device’s current and voltage. Nitrile performed the best with an electrical output of 125 V and ~12.5 µA for the open-circuit voltage and short-circuit current, respectively. Finally, a theft alarm system was built to harvest energy from the drawer’s push–pull action. The TENG in the drawer was designed to avoid theft by sending a message when opened. The device illuminates an LED, Lumex-LCD-S2X1C50TR, and triggers a buzzer sound to alert the owner via a mobile phone through the use of an Arduino device.

## Data Availability

The data provided in this work are accessible on request from the corresponding author.

## Supplementary Materials

The following supporting information can be downloaded at: https://www.mdpi.com/article/10.3390/mi14030592/s1. Figure S1: The stability analysis for the short duration of 100 s; Figure S2: The output voltage of the SM-TENG with different electrode gap distances; Figure S3: Electrical output voltage of forward and backward sliding at low speed.
