# Supplementary material for: Self-Powered Triboelectric Nanogenerator for Security Applications
Source: Micromachines (Basel). 2023 Mar 1;14(3):592. doi: 10.3390/mi14030592 (PMC10056479; doi:10.3390/mi14030592)
Supplement: Supplementary file 1 [file micromachines-14-00592-s001.zip › micromachines-2189280-supplementary.pdf]

Supplementary Materials

Self-Powered Triboelectric Nanogenerator for Security Applications

Prabavathi Munirathinam and Arunkumar Chandrasekhar *

Nanosensors and Nanoenergy Lab, Sensor Systems Lab, Department of Sensors and Biomedical Technology, School of Electronic Engineering, Vellore Institute of Technology, Vellore 632014, Tamil Nadu, India
* Correspondence: arunkumar.c@vit.ac.in

It is proven that the output performance is stable for the 10 days of continuous measurement in Figure 5a. This performance shows that the device can generate output for a long period of time, which is highly important in real-time applications. The stability of the device was also tested for the short duration of 100 seconds which is shown in Figure S1. The SM-TENG generates the best electrical output for the 1 cm and the output is shown in Figure S2. This shows the gap distance between the electrodes has significant changes in the triboelectric performance. The optimum sensor design structure of the proposed sensor is 1.5 cm * 1 cm. Figure S3 shows the voltage response of forward and backward slow sliding motion.

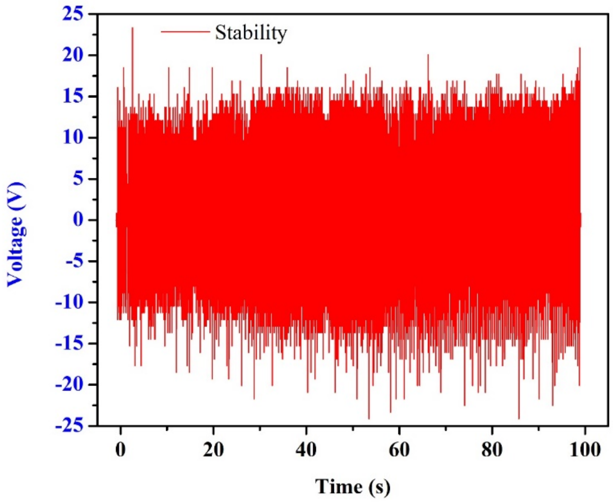

Figure S1. The stability analysis for the short duration of 100 seconds.

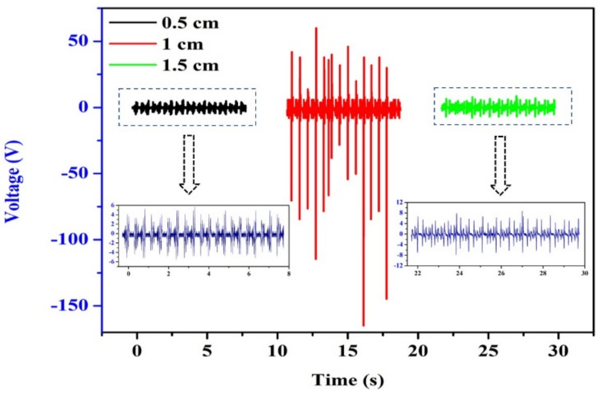

Figure S2: The output voltage of the SM-TENG with different electrode gap distances.

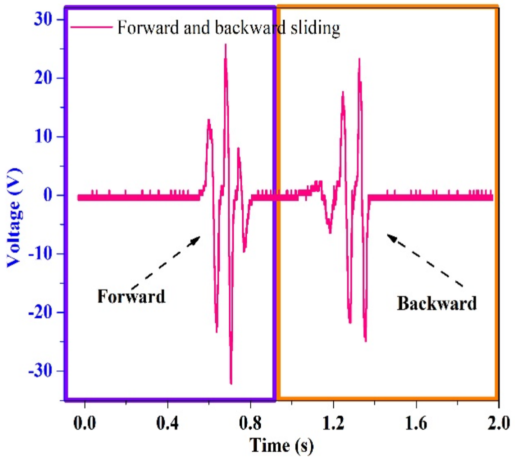

Figure S3: Electrical output voltage of forward and backward sliding at low speed.
